# Role of the SOX family in non-small cell lung cancer: Molecular mechanisms and therapeutic implications (Review)

**DOI:** 10.3892/or.2025.9024

**Published:** 2025-11-18

**Authors:** Kaiwei Wang, Yaoqi Li, Zhening Guo, Lin Song, Xiaoliang Ding, Linsheng Liu, Tao Hu, Yicong Bian, Chenrong Huang, Liyan Miao

**Affiliations:** 1 Department of Pharmacy, The First Affiliated Hospital of Soochow University, Suzhou, Jiangsu 215006, P.R. China; 2 Institute for Interdisciplinary Drug Research and Translational Sciences, Soochow University, Suzhou, Jiangsu 215006, P.R. China; 3 College of Pharmaceutical Sciences, Soochow University, Suzhou, Jiangsu 215123, P.R. China; 4 Department of Pharmacy, The Second Affiliated Hospital of Soochow University, Suzhou, Jiangsu 215004, P.R. China

**Keywords:** SOX family, non-small cell lung cancer, molecular mechanism

## Abstract

Non-small cell lung cancer (NSCLC), accounting for >85% of LC cases, remains a therapeutic challenge due to its low 5-year survival rate, tumor heterogeneity and drug resistance. The SRY-related high-mobility group-box (SOX) family comprises transcription factors involved in the initiation and progression of NSCLC. These factors regulate epithelial-mesenchymal transition and angiogenesis, interact with epidermal growth factor receptor/KRAS pathways to influence tumor invasion and promote chemotherapy resistance by sustaining tumor stemness. The present review aimed to summarize the expression patterns, molecular mechanisms and clinical relevance of SOX family members (such as SOX2, SOX4 and SOX9) in NSCLC, as well as their potential as diagnostic biomarkers and therapeutic targets, and the application of emerging technology in elucidating their functions. The present review aimed to provide a theoretical foundation for precision diagnostics and therapeutics to foster more effective NSCLC treatment.

## 1. Introduction

As one of the most prevalent and deadly malignancies worldwide, non-small cell lung cancer (NSCLC) accounts for >85% of LC cases (1–3). NSCLC exhibits notable molecular heterogeneity, with key driver gene mutations including epidermal growth factor receptor (EGFR), kirsten ratsarcoma viral oncogene homolog (KRAS) and anaplastic lymphoma kinase. These mutations play a crucial role in determining treatment strategies and prognosis for patients (4,5). Although targeted therapies and immunotherapies have notably improved survival in some patients, the 5-year survival rate for NSCLC remains <20%, particularly in advanced-stage cases, where tumor heterogeneity, metastatic potential and drug resistance pose notable therapeutic challenges (6,7). Therefore, investigating the mechanisms underlying NSCLC development and progression, as well as identifying novel therapeutic targets, remains a key focus.

Members of the SOX family are evolutionarily conserved transcription factors that regulate gene expression by binding DNA, playing a central role in embryonic development, cell fate determination and the maintenance of stem cell pluripotency (8,9). Studies have revealed the abnormal expression of SOX family members such as SOX2, SOX4 and SOX9 in various solid tumors, including colorectal, breast and liver cancers (10–12). These factors contribute to tumor malignancy by regulating cancer stemness, epithelial-mesenchymal transition (EMT), metabolic reprogramming and microenvironment remodeling (13,14). Particularly in NSCLC, SOX proteins exhibit dual regulatory functions: Certain members (SOX2) may promote chemotherapy resistance by maintaining cancer stem cell properties, whereas others (SOX17) serve as tumor suppressors by inhibiting oncogenic signaling pathways (15). This functional diversity suggests the SOX family forms a complex regulatory network in NSCLC, the precise mechanisms of which require systematic elucidation. However, expression patterns of different SOX members across NSCLC subtypes and molecular classifications, as well as their clinical significance, remain incompletely understood. Moreover, the interplay between their downstream regulatory networks and epigenetic modifications requires further investigation. Furthermore, translating the molecular functions of SOX proteins into clinical applications, such as their development as biomarkers or therapeutic targets, remains a notable challenge.

With advancements in high-throughput sequencing technologies, researchers can gain a more detailed understanding of genomic alterations in SOX family members, offering insight into the complexity of NSCLC. Single-cell RNA sequencing provides an opportunity to analyze intratumoral heterogeneity more precisely, which is key for identifying the specific functions and roles of SOX family members (16). Notably, intervention strategies targeting SOX family members are also evolving. Techniques based on small interfering (si)RNA or clustered regularly interspaced short palindromic repeats/CRISPR-associated protein 9 (CRISPR/Cas9) have been explored to suppress the expression or function of SOX proteins with the aim of achieving therapeutic effects (17). These emerging therapeutic approaches not only expand understanding of potential NSCLC treatments but also offer new perspectives for future personalized medicine.

The present study aimed to summarize the expression patterns, molecular mechanism and clinical relevance of SOX protein in NSCLC, with emphasis on SOX2, SOX4 and SOX9 (implicated in critical oncogenic processes, including cell proliferation, epithelial-mesenchymal transition, stemness maintenance, and chemoresistance (18–21), their role in tumor progression, stem cells and drug resistance, as well as their potential as diagnostic biomarkers and therapeutic targets. Comprehensive understanding of the SOX family in NSCLC may drive the development of more personalized and effective treatments, ultimately improving patient outcomes and quality of life.

## 2. Expression characteristics of SOX family members in NSCLC

**Figure 1.**
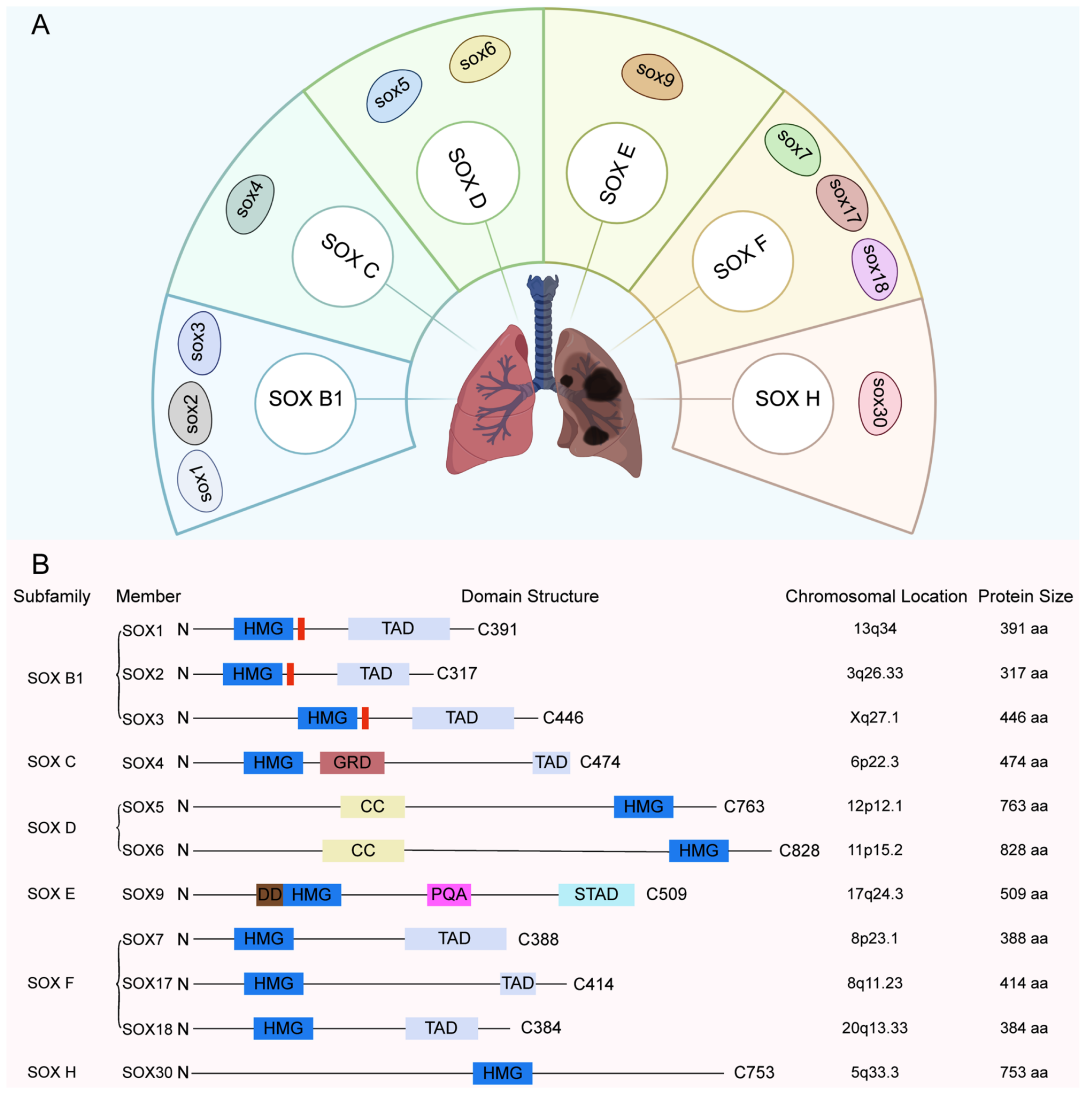
Classification and structure of the SOX family in non-small cell lung cancer. (A) Classification of the SOX family in non-small cell lung cancer. (B) Structure of SOX family. HMG, high mobility group; TAD, transactivation domain; GRD, Glycine-Rich domain; CC, Coiled-coil domain; DD, dimerization domain; PQA, Proline-Glutamine-Alanine-rich domain; aa, amino acid.

The SOX gene family encodes a series of transcription factors characterized by a highly conserved domain and is classified into ten subgroups (A-J) based on protein homology (22). In LC research, the subgroups most frequently involved include B1, C, D, E, F and H (Fig. 1). These transcription factors bind DNA through their high mobility group box domains and regulate gene expression, thereby serving pivotal roles in physiological processes such as embryonic development, cell fate determination and differentiation. For example, SOX4, a member of the C subgroup, serves as a key mediator of TNF-α-induced transformation of fibroblast-like synoviocytes in the pathological progression of arthritis. It exerts its regulatory effects by interacting with the transcription factor v-rel reticuloendotheliosis viral oncogene homolog A/p65) in the NF-κB signaling pathway, thereby cooperatively modulating the expression of downstream genes under TNF signaling (23). Similarly, SOX3, a member of the SOXB1 subfamily, is broadly expressed during embryogenesis, neurogenesis and gonadal development in *Misgurnus* (loach), and this expression pattern is conserved throughout vertebrate evolution (24). In addition, the expression of multiple SOX genes is observed in stem, undifferentiated progenitor and differentiated cells with neuro-sensory characteristics in cnidarians, further supporting the ancient evolutionary conservation of the SOX gene family in developmental biology (22). Different SOX subgroups demonstrate functional specificity. For example, SOX5 and SOX6 from the SOXD subgroup are involved in transcriptional regulation during embryonic development, neural growth and chondrogenesis (25). A member of the SOXE subgroup, SOX9, serves a key role in sex determination and gonadal development in various species (26). Notably, the majority of SOX family members exhibit aberrant expression in NSCLC, influenced by mechanisms including gene mutations, DNA methylation and regulation by microRNAs (miRs; Table I) (27). These dysregulated SOX genes have notable effects on the initiation and progression of NSCLC. Elucidating the functions and regulatory mechanisms of these SOX genes will not only deepen understanding of NSCLC pathogenesis but may provide potential targets for the development of novel targeted therapeutic strategies.

### SOX2

The SOX2 gene is located on human chromosome 3q26.3-q27 (Fig. 1B). As a critical member of the SOX gene family, SOX2 is associated with the initiation and progression of NSCLC, exhibiting high expression in lung squamous cell carcinoma. A fluorescence *in situ* hybridization analysis of 447 surgically resected NSCLC specimens revealed SOX2 gene copy number gain in 23.6% of cases (28). This genomic alteration is associated with a history of smoking, the squamous cell carcinoma histological subtype and copy number gains of other genes, including PIK3CA, fibroblast Growth Factor Receptor 1 and v-raf murine sarcoma viral oncogene homolog B1) (28). In a separate study involving 60 cases of oral squamous cell carcinoma, SOX2 expression was detected in 63% of tumors (29). Notably, SOX2 levels are inversely associated with tumor grade and positively associated with progression-free survival (29). Further investigations have suggested that SOX2 upregulation in NSCLC may influence tumorigenesis through multiple molecular pathways (30,31). For example, in NSCLC patients with non-adenocarcinoma subtypes, the expression of transglutaminase 2 is linked to tumor recurrence and disease-free survival, and SOX2 is hypothesized to regulate this (30). Additionally, SOX2 promotes tumor cell proliferation and survival by upregulating cyclin D1 and activating the Wnt/β-catenin pathway, contributing to aggressive tumor behavior in NSCLC (31). Moreover, as a risk factor for NSCLC, smoking may contribute to altering the expression of long non-coding RNAs (lncRNAs), which functionally interact with SOX2 (32). This implies SOX2 may exert its oncogenic effects through mechanisms involving lncRNA dysregulation. Collectively, these findings highlight the key role of SOX2 in the development and progression of NSCLC, particularly squamous cell carcinoma, and support its potential as a promising candidate for diagnostic and therapeutic targeting in clinical settings.

### SOX4

SOX4 plays a pivotal role in the initiation and progression of NSCLC. Studies have demonstrated that SOX4 is significantly upregulated in NSCLC tissue and serves as an independent prognostic marker, underlining its key role in malignant tumor progression (33,34). At the molecular level, SOX4 promotes NSCLC cell migration, invasion and EMT by upregulating B cell-specific MLV integration site-1 (BMI1) expression (35). BMI1 induces the ubiquitination of histone H2A, which suppresses the expression of zinc finger protein 24 and decreases the secretion of vascular endothelial growth factor A, thereby promoting tumor angiogenesis and providing support for tumor cell proliferation and metastasis. Moreover, SOX4 regulates cell processes, including proliferation, survival and migration of NSCLC cells, through interactions with signaling pathways such as PI3K/AKT (36,37). Furthermore, the hypoxia-sensitive lncRNA CASC15 can promote the occurrence of LC by regulating the SOX4/β-catenin axis. Under hypoxic conditions, CASC15 transcription is activated, promoting the expression of SOX4, stabilizing the β-catenin protein and ultimately enhancing NSCLC cell proliferation and migration abilities (38). Meanwhile, circular (circ)RNA circ_0020714 serves as an endogenous miR-30a-5p sponge, enhancing the expression of SOX4 and promoting immune escape and anti-PD-1 resistance in patients with NSCLC (39). These findings highlight the multifaceted role of SOX4 in NSCLC pathogenesis and progression, making it a potential target for therapeutic interventions.

### SOX9

EGFR and KRAS mutations are common oncogenic drivers in lung adenocarcinoma and serve a crucial role in tumorigenesis (40). SOX9 expression is elevated in lung adenocarcinoma, particularly those with KRAS mutations, and mediates Notch1-induced EMT. A previous study demonstrated higher SOX9 mRNA in KRAS-compared with EGFR-mutant tumors (41). This suggests SOX9 acts downstream of Notch in KRAS-driven NSCLC, promoting invasion and metastasis, with potential indirect antagonism to EGFR signaling due to mutual exclusivity of KRAS/EGFR mutations (41). Furthermore, SOX9 is essential for KRAS-driven lung adenocarcinoma progression. Loss of SOX9 decreases tumor burden, extends survival and enhances anti-tumor immunity by increasing levels of tumor-infiltrating dendritic cells and CD8^+^ T cells (42). Although direct evidence regarding the interaction between SOX9 and the EGFR/KRAS pathways in lung adenocarcinoma remains limited, studies in other types of cancer suggest that SOX9 may regulate the biological behavior of lung adenocarcinoma cells through crosstalk with these signaling pathways (42–44). For example, aberrant activation of EGFR or KRAS may regulate SOX9 expression or activity via downstream signaling cascades, thereby affecting cell processes such as proliferation, differentiation, migration and invasion (42–44). Additionally, in colorectal cancer, SOX9 activates the canonical Wnt/β-catenin pathway by promoting β-catenin stability, nuclear translocation, and transcription of Wnt components like FZD7 and LRP6, which drives tumor growth, metastasis, and stem cell-like properties (45,46). In pancreatic cancer, the oncogene KRAS induces the expression of SOX9 at both the mRNA and protein levels, including its phosphorylated form, thereby promoting SOX9 nuclear translocation and transcriptional activity (47,48). The transforming growth factor-β-activated kinase 1/IκBα/NF-κB signaling pathway is involved in the regulation of SOX9 by KRAS. In addition, SOX9 modulates the expression of mediator of DNA damage checkpoint 1and minichromosome maintenance complex components, which are associated with tumor cell proliferation, invasion and metastasis (47). Furthermore, in the context of KRAS mutations, ectopic expression of SOX9 in acinar cells synergizes with oncogenic KRAS to markedly accelerate the formation of precancerous lesions (48). In urothelial carcinoma, the activation of EGFR can upregulate the expression of SOX9 via the ERK signaling pathway, thereby promoting tumor occurrence; the EGFR-ERK-SOX9 signaling cascade mechanism suggests that a similar regulatory pathway may also exist in EGFR-mutated NSCLC (44). Elucidating the mechanistic interplay between SOX9 and the EGFR/KRAS pathways in lung adenocarcinoma holds promise for identifying novel therapeutic targets and developing precision medicine strategies.

### Other SOX genes

**Figure 2.**
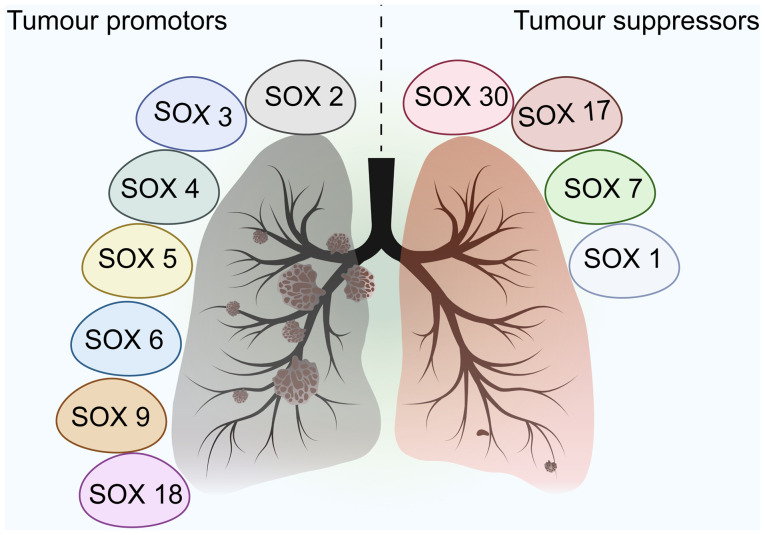
Members of the SOX family exhibit functional diversity in the occurrence and development of non-small cell lung cancer. Certain members can affect the proliferation, apoptosis, invasion and metastasis abilities of tumor cells by regulating key signaling pathways, thereby exerting tumor-suppressing or -promoting effects.

In addition to SOX2, SOX4, and SOX9, other members of the SOX family regulate the initiation and progression of NSCLC (Fig. 2). For example, SOX5 is highly expressed in NSCLC and may serve as a supportive prognostic marker for the diagnosis of NSCLC (49,50). In a cohort of 90 patients with lung adenocarcinoma, immunohistochemical analysis revealed elevated SOX5 expression in tumor compared with adjacent non-tumor tissues. High SOX5 levels were significantly associated with advanced clinical stage, lymph node metastasis and reduced overall survival. Multivariate Cox regression confirmed SOX5 as an independent prognostic factor for poor survival (51). Functional assays show SOX5 promotes EMT, enhancing invasion and migration, while knockdown inhibits these processes *in vitro* and *in vivo* (52). SOX5 is also aberrantly upregulated in NSCLC cell lines, where it promotes proliferation, migration, invasion and EMT via interaction with YAP1. Knockdown of SOX5 suppresses tumor growth and metastasis, and ncRNAs such as miR-143-3p that target SOX5 inhibit cancer progression, underscoring its prognostic significance (51,53). Additionally, elevated SOX18 expression is associated with poor prognosis, and knockdown of SOX18 notably impairs cellular migratory capacity (54,55). In a cohort of 198 NSCLC cases, SOX18 was expressed in the nuclei and cytoplasm of cancer cells in 94.4 and 47% of the NSCLC cases, respectively (54). SOX18 mRNA levels are lower in NSCLC than in non-malignant lung tissue, but protein levels are higher. Cytoplasmic SOX18 expression is associated with poor patient outcome, and nuclear SOX18 is positively associated with Ki-67 proliferation index, suggesting its role in tumor progression and potential as a prognostic biomarker (54). Although the specific role of SOX3 in NSCLC remains largely unexplored, aberrant expression of SOX3 has been reported in other malignancies such as osteosarcoma and breast cancer (56,57). In these contexts, SOX3 dysregulation is implicated in tumor development and progression, potentially via its influence on apoptosis, migration and proliferation (56,58,59). To elucidate its potential role in NSCLC, machine learning approaches may be employed to integrate multi-omics datasets (such as The Cancer Genome Atlas/Gene Expression Omnibus) for predicting SOX3 interactions with pathways such as Wnt or EGFR. Furthermore, artificial intelligence-driven clustering of SOX expression with tumor mutational burden (TMB) and immune signatures may help identify resistance patterns or novel biomarkers, while bioinformatics analysis of SOX3 methylation may demonstrate diagnostic value (60,61). Furthermore, SOX7 and SOX17 are notably associated with prognosis in lung adenocarcinoma (60,62,63). Patients with high expression of SOX7 and SOX17 exhibit better overall survival, suggesting these genes may serve as potential prognostic biomarkers (60,62,63).

## 3. Molecular mechanisms of SOX family in regulating NSCLC

**Figure 3.**
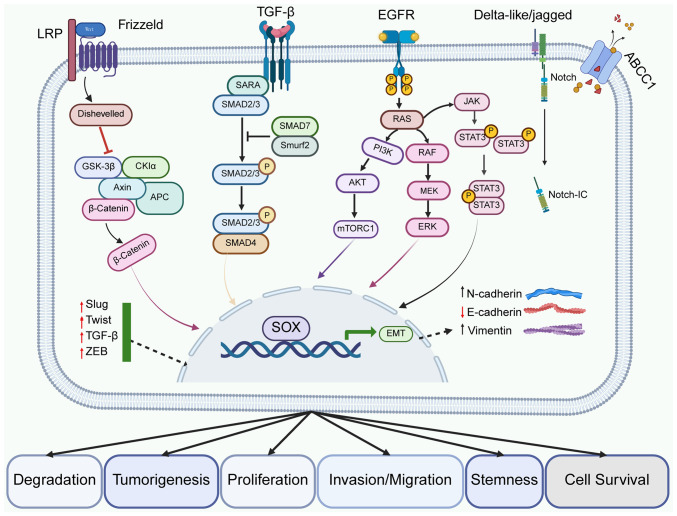
Members of the SOX family play important roles in multiple biological processes such as cell proliferation, apoptosis, migration, stem maintenance and drug tolerance of NSCLC by interacting with key signaling pathways such as Wnt/β-catenin, TGF-β and EGFR. The abnormal regulation of these signaling pathways not only affects the basic behavioral characteristics of tumor cells, but may also lead to changes in the response of NSCLC to treatment and disease progression. NSCLC, non-small cell lung cancer; LRP, low-density lipoprotein receptor-related protein; ABCC, ATP-binding cassette, sub-family C; SARA, Smad-anchor for receptor activation; GSK, glycogen synthase kinase; CKI, Casein Kinase I; ZEB, Zinc Finger E-Box Binding Homeobox; EMT, Epithelial-Mesenchymal Transition; IC, Intracellular Domain.

The SOX family is involved and development of NSCLC by regulating biological behaviors, including cell proliferation, apoptosis, invasion and metastasis, as well as the maintenance of tumor stem cells (Fig. 3).

### Role of the SOX family in NSCLC cell proliferation (SOX2, SOX4, SOX17)

Studies have demonstrated that certain SOX members actively promote NSCLC cell proliferation (35,63,64). For example, SOX2 facilitates cell proliferation and survival by regulating cell cycle progression and DNA damage repair (64). In models of radioresistant NSCLC cell lines, SOX2 expression is markedly upregulated (65–67). Overexpression of SOX2 enhances resistance to radiotherapy and improves DNA repair capacity, thereby promoting proliferation (68). Conversely, SOX2 knockdown impairs these functions and suppresses cell proliferation (69). Similarly, SOX4 supports NSCLC cell proliferation indirectly by promoting migration, invasion and EMT (21). Analyses of patients with NSCLC has revealed significantly elevated SOX4 expression in tumor tissue, identifying it as an independent prognostic marker (35). However, not all SOX family members serve as oncogenes. For example, SOX17 is downregulated in lung adenocarcinoma, and its upregulation suppresses NSCLC cell proliferation, suggesting a potential tumor-suppressive function (63).

### Role of the SOX family in NSCLC cell apoptosis (SOX4, SOX6, SOX9)

The SOX family serves a key role in the regulation of apoptosis in NSCLC cells, constituting an essential component of its involvement in tumor pathophysiology (70). SOX6 not only inhibits the proliferation of lung adenocarcinoma cells but also promotes apoptosis by modulating the expression of key proteins such as p53, cyclin-dependent kinase inhibitor 1A, cyclin D1 and β-catenin, thereby impacting cell cycle control and apoptosis-associated signaling pathways (71). In studies of erlotinib resistance in NSCLC cells, OTU domain-containing 1 (OTUD1) enhances cellular sensitivity to erlotinib by inhibiting YAP1 nuclear translocation, accompanied by the inactivation of the SOX9/secreted phosphoprotein 1 pathway (72,73). Notably, overexpression of SOX9 reverses the sensitizing effects of OTUD1, indicating a role for SOX9 in apoptosis and drug resistance mechanisms in NSCLC (72). In addition, SOX9, together with STAT3, forms a differential regulatory network that may contribute to erlotinib resistance by modulating cellular proliferation and survival signaling pathways (73). Furthermore, SOX-associated signaling pathways are also involved in the regulation of apoptosis. For example, in endometrial cancer cells, propofol inhibits proliferation, migration and invasion, and promotes apoptosis by downregulating SOX4 expression, which is associated with inactivation of the Wnt/β-catenin signaling pathway, indicating that the Wnt/β-catenin-SOX4 axis may be involve in the apoptosis of NSCLC cells (74). Furthermore, SOX4 affects the proliferation and apoptosis of NSCLC cells by regulating cell cycle-associated proteins (33). For example, SOX4 may alter the expression of proteins such as Cyclin D1, promoting the transition of cells from the G1 to the S phase, thereby promoting cell proliferation. By regulating the expression of apoptosis-associated proteins such as Bax and Bcl-2, SOX4 affects the occurrence of apoptosis (33). circ_0089823 affects cell proliferation and apoptosis by regulating SOX4. Knockdown of circ_0089823 inhibits the proliferation of NSCLC cells, induces cell cycle arrest and apoptosis (75). Overexpression of circ_0089823 promotes malignant behavior such as cell proliferation, and SOX4 is positively regulated by circ_0089823. Silencing SOX4 can counteract the effect of overexpression of circ_0089823 on NSCLC cells (75).

### Role of SOX family in NSCLC cell invasion and metastasis (SOX1, SOX4, SOX30)

In the invasion and metastasis of NSCLC, multiple signaling pathways and molecular mechanisms are associated with the SOX family (76). For example, in LC, SOX1 expression is markedly downregulated, which contributes to tumor initiation and progression. SOX1 suppresses the malignant progression of NSCLC by inhibiting the hairy and enhancer of split 1 factor, thereby suppressing anchorage-independent growth, invasion and metastatic behavior (77). Furthermore, SOX4 promotes tumor invasion and metastasis by upregulating LEM domain containing 1, which activates the PI3K/Akt signaling pathway in colorectal cancer, implying that SOX4 may facilitate NSCLC progression through a similar mechanism (78). In addition, miR-363-3p can inhibit the migration, invasion and EMT of NSCLC cells by targeting SOX4. The overexpression of miR-363-3p inhibits cell migration and invasion, while knockdown of miR-363-3p shows the opposite effect (79). Further studies have found that miR-363-3p directly binds the 3′-untranslated region of SOX4 and negatively regulates its expression, and neural precursor cell expressed developmentally downregulated 9 or SOX4 knockdown can salvage the translocation-promoting effect of antagomiR-363-3p (80–82). In lung adenocarcinoma, SOX30 inhibits Wnt signaling by directly suppressing the transcription of β-catenin, thereby blocking cell migration and invasion. High SOX30 expression is associated with better patient prognosis (76,83). Additionally, interactions between SOX genes and other molecular regulators influence NSCLC metastasis (76). For example, elevated expression of lncRNA KCNQ1OT1 is notably associated with tumor size, TNM stage and lymph node metastasis in NSCLC. This lncRNA promotes proliferation, migration and invasion via the miR-129-5p/Jagged1 pathway. While SOX genes are not directly involved in this pathway, the associated signaling networks suggest potential regulatory interplay (84). Moreover, miR-548l suppresses NSCLC cell migration and invasion by targeting the AKT1 signaling pathway, indicating SOX genes may participate in coordinated regulation of invasion and metastasis through crosstalk with such pathways (85).

### Role of the SOX family in maintaining NSCLC stemness (SOX2, SOX9)

The SOX family is key for the maintenance of cancer stemness in NSCLC cells (86). Tumor stemness refers to the capacity of cancer cells for self-renewal and differentiation, and is associated with tumorigenesis, progression, recurrence and therapy resistance (87). SOX2 has been shown to enhance stemness in NSCLC by modulating aerobic glycolysis. In both NSCLC tissue and cell lines, SOX2 and protein disulfide isomerase family A, member 6 (PDIA6) are highly expressed and functionally related. Knockdown of PDIA6 decreases expression of stemness-associated markers and impairs spheroid-forming ability, while PDIA6 overexpression enhances these characteristics (88). This effect is reversible with the glycolysis inhibitor 2-deoxy-D-glucose, indicating that the SOX2/PDIA6 pathway promotes NSCLC cell stemness via modulation of glycolytic metabolism (88). SOX9 also contributes to NSCLC stemness. Under hypoxic conditions, SOX9 undergoes lactylation, which enhances stemness, migration, and invasion (89). Inhibition of glycolysis can reverse these effects, suggesting targeting hypoxia-associated SOX9 regulation may offer a promising therapeutic strategy for NSCLC (89).

### Role of the SOX family in drug resistance in NSCLC (SOX2, SOX4)

The SOX family serves a notable role in the development of drug resistance in NSCLC (18,90). For example, SOX2 is associated with resistance to paclitaxel and platinum-based chemotherapeutics. In studies using A549 NSCLC cell lines (68,91), SOX2 enhances resistance to paclitaxel by promoting the transcription of chloride voltage-gated channel 3 (ClC-3). Knockdown of either SOX2 or ClC-3 significantly decreases drug resistance (68). SOX2 overexpression attenuates the Wnt/β-catenin signaling activity in both lung adenocarcinoma A549 cells and cisplatin-resistant counterpart A549/DDP cells through upregulation of GSK3β, a key negative regulator of this pathway (91). Additionally, SOX2 modulates resistance to cisplatin through the AP endonuclease 1 (APE1) signaling pathway, and silencing SOX2 restores cisplatin sensitivity (69). Other SOX family members are also implicated in resistance mechanisms. For example, upregulation of SOX4 is associated with chemoresistance in NSCLC (92,93). miR-129-2 enhances chemosensitivity by targeting SOX4 and inducing apoptosis, indicating modulation of SOX4 levels influences drug response (92). In-depth exploration of the molecular mechanisms underlying SOX-mediated resistance in NSCLC may provide valuable insights for the development of novel therapeutic strategies.

## 4. Targeting SOX genes for personalized NSCLC therapy

### Prospects for SOX family in personalized NSCLC treatment

Given the critical role of the SOX family in NSCLC development, individualized therapies targeting these genes hold promise. Analyzing the expression profiles, mutation status and associated signaling pathways of SOX genes in tumors enables precise patient stratification, providing a basis for tailoring individualized treatment strategies. For example, in patients with high SOX gene expression associated with poor prognosis, targeted interventions [such as small-molecule inhibitors, RNA interference (RNAi) or immunotherapy-based combination strategies] can be developed or selected to suppress SOX gene function. Integrating these findings with clinical features and molecular markers may further refine treatment regimens and enhance therapeutic efficacy (17). Moreover, advancements in drug development technologies have facilitated the creation of SOX-specific targeted therapies. These drugs precisely target SOX genes or their related signaling pathways, minimizing toxicity to normal cells while improving treatment safety and effectiveness (94,95). Additionally, integrating genetic backgrounds and lifestyle factors contributes to more precise individualized therapies, improving NSCLC prognosis.

### NSCLC treatment strategies targeting SOX genes

Targeting SOX genes represents a promising therapeutic strategy for NSCLC, aiming to suppress tumor growth by modulating SOX gene expression or activity. RNAi has been employed to silence SOX gene expression, altering the biological behavior of NSCLC cells. For example, in cisplatin-resistant NSCLC cell lines, SOX2 upregulation promotes resistance via APE1 signaling. Small interfering (si)RNA-mediated SOX2 knockdown (siSOX2) inhibits colony formation, decreases cell viability, enhances apoptosis and restores cisplatin sensitivity. Combined siSOX2 and cisplatin treatment inhibits tumor progression *in vitro*, with low SOX2 expression linked to better patient survival (69). Silencing SOX4 has been shown to inhibit proliferation, migration and invasion of osteosarcoma cells, while also inducing apoptosis (96). These findings suggest that targeting SOX4 may similarly regulate progression of NSCLC (96). Additionally, several novel small-molecule compounds have been developed to target SOX proteins: Nobiletin, a small molecule, binds to the transcription factor SOX5, exhibiting synergistic cytotoxic effects when combined with doxorubicin in SOX5-overexpressing cells (97). This highlights a potential combination therapy approach for NSCLC. Furthermore, the development of targeted drugs based on signaling pathways involving SOX genes has become a focal point of research (98–100). In NSCLC, histone deacetylase 7 (HDAC7) promotes tumor proliferation and metastasis by activating the β-catenin/FGF18 pathway, suggesting that targeting HDAC7 or its associated signaling cascades may serve as a novel therapeutic strategy (98). Moreover, CRISPR/Cas9 gene-editing technology offers a tool to modify SOX genes (101). Although this approach currently faces technical challenges (off-target effects, delivery challenges, editing efficiency) (102,103) for clinical translation, it holds promise for personalized cancer therapies. To the best of our knowledge, no clinical trials involving siRNA therapy targeting SOX genes have been reported to date. The majority of research remains at the preclinical stage (69,96,97), encompassing *in vitro* studies, animal models and the optimization of small molecule delivery systems. This delay in clinical translation may be attributed to challenges associated with siRNA delivery, including issues of stability, targeting efficiency and off-target effects (104,105). Although clinical research on siRNA therapies targeting SOX genes in LC remains in its early stages, siRNA technology has demonstrated notable clinical progress across various oncological indications, providing robust support for its potential application in malignancy. For example, NBF-006, a lipid nanoparticle-formulated siRNA targeting the KRAS G12D mutation (present in ~25% of patients with NSCLC), has been evaluated in a Phase I clinical trial (106) for KRAS-mutant NSCLC and pancreatic and colorectal cancer. This trial has demonstrated sustained KRAS silencing within tumors and improved safety and tolerability compared with small-molecule inhibitors (106). In glioblastoma multiforme (GBM), NU-0129, a gold nanoparticle-conjugated siRNA targeting bcl-2-like protein 12, an anti-apoptotic factor upregulated in GBM, achieves localized tumor delivery via convection-enhanced delivery. In patients with recurrent GBM, it induces tumor cell apoptosis without evidence of neurotoxicity, progressing to a Phase Ib expansion study (107). Additionally, CALAA-01, a cyclodextrin-based nanoparticle delivery system to target the M2 subunit of ribonucleotide reductase, was investigated in a Phase I trial (108) across various solid tumors, including ovarian and peritoneal cancer. Tumor biopsy confirmed activation of the RNAi mechanism, with clinical outcomes indicating partial responses in 11% of patients and disease stabilization in 48% (108,109). Collectively, these early-phase clinical studies validate the feasibility and therapeutic potential of siRNA-based approaches in multiple types of cancer. They also provide a key foundation for the development of novel RNAi strategies targeting the SOX pathway, offering promise for advancing precision therapeutics in NSCLC and optimization and innovation in its treatment paradigm.

### Integration of the SOX family with immunotherapy in NSCLC

Immunotherapy harnesses the host immune system to target tumor cells, and SOX genes may play a pivotal regulatory role within the tumor immune microenvironment. The integration of SOX family-targeted therapies with immunotherapy offers new possibilities for the treatment of NSCLC. Expression of certain SOX genes is notably associated with the quantity and type of tumor-infiltrating immune cells, suggesting their potential influence on immunotherapy efficacy (39,110). For example, expression of SOXF family genes is positively associated with CD4^+^ T cell infiltration, a characteristic that may modulate tumor response to immunotherapy (60). In NSCLC, SOX2 upregulates IL6 via FOS-like antigen 2, promoting inflammation and metastasis while suppressing CD8^+^ T cell infiltration via cyclic GMP-AMP synthase/STING degradation (111). SOX2/SOX9 enable natural killer cell evasion by downregulating major histocompatibility complex class I markers in cancer cells. SOX expression is associated with checkpoints and TMB, predicting immunotherapy response. Targeting SOX may enhance PD-1 blockade and radioimmunotherapy (112). Furthermore, targeting SOX genes can enhance the effectiveness of immunotherapy in NSCLC. For example, the use of proteolysis-targeting chimeras (PROTACs) to degrade EGFR L858R has been shown to downregulate PD-L1 and indoleamine 2,3-dioxygenase 1 protein levels, thereby amplifying anti-tumor immune responses and providing an approach to NSCLC immunotherapy (113). Additionally, combining immune checkpoint inhibitors with modulators targeting SOX gene-associated signaling pathways in NSCLC immunotherapy has also shown enhanced efficacy (114). For instance, tumor-intrinsic SOX2 signaling in NSCLC promotes the recruitment of regulatory T cells (Tregs) by upregulating CCL2, thereby mediating resistance to ICIs. Depletion of Tregs or inhibition of the SOX2 pathway restores T cell infiltration and markedly suppresses tumor growth, suggesting that targeting the SOX2 pathway may synergistically enhance the therapeutic efficacy of ICIs (110). This strategy may optimize the tumor immune microenvironment and improve therapeutic outcomes, warranting further exploration and validation.

## 5. Conclusion

NSCLC, the predominant subtype of LC, poses notable clinical challenges due to its molecular heterogeneity, drug resistance and low survival rate (115,116). The present review summarized the key role of the SOX family of transcription factors in the initiation, progression and treatment of NSCLC. Key members such as SOX2, SOX4 and SOX9 notably influence the malignant progression of NSCLC by regulating processes including tumor stemness, EMT, cell proliferation, apoptosis, invasion, metastasis and drug resistance (35,89,117). For example, SOX2 sustains tumor stemness to promote chemotherapy resistance, SOX4 modulates EMT and angiogenesis and SOX9 collaborates with EGFR/KRAS signaling pathways to drive tumor invasion. Conversely, other SOX family members, such as SOX17, exhibit tumor-suppressive potential, underscoring the dual functionality within the SOX family (63).

Molecular mechanistic studies have revealed that SOX genes participate in NSCLC pathogenesis through intricate signaling networks (such as Wnt/β-catenin and PI3K/AKT) and epigenetic regulation (118,119). Clinically, dysregulated SOX expression is associated with patient outcomes, highlighting the potential of SOX family members as diagnostic biomarkers and therapeutic targets (76,120). In parallel, certain emerging technologies (such as single-cell sequencing and CRISPR/Cas9) provide novel tools to determine the roles of the SOX family, while the development of SOX-targeted therapy (including RNAi and PROTACs) and their integration with immunotherapy offer new avenues for precision NSCLC therapy. However, clinical translatability remains a challenge. siRNA-based RNAi, for example, excels in sequence-specific SOX silencing, with preclinical NSCLC models showing decreased tumor growth and stemness following SOX2 or SOX9 knockdown (69,121,122). However, its limitations include poor *in vivo* stability (rapid nuclease degradation and renal clearance, often limiting half-life to <24 h), inefficient cell uptake due to negative charge, endosomal entrapment and potential off-target effects or immune activation in the fibrotic NSCLC tumor microenvironment (123–125). PROTACs induce sustained ubiquitin-mediated degradation of SOX proteins, targeting undruggable surfaces without relying on enzymatic pockets, potentially overcoming compensatory upregulation seen in SOX family members. In NSCLC, PROTACs have shown promise against transcription factors such as STAT3 analogs, but face hurdles in E3 ligase selectivity (risking off-target toxicity) and dependency on endogenous ubiquitin machinery (126,127). CRISPR/Cas9 offers permanent genomic editing of SOX loci, bypassing delivery instability by enabling knockout or base editing in preclinical patient-derived tumor xenograft models (128–130), where SOX9 ablation halts metastasis more durably than siRNA. However, its application is constrained by vector-associated immunogenicity, potential off-target genomic alterations and ethical and regulatory barriers (17,131,132).

Taken together, the present findings highlight the promise and the limitations of SOX-targeted strategies in NSCLC. Future studies should focus on optimizing delivery platforms, enhancing therapeutic specificity and integrating SOX-based interventions with existing targeted therapies and immunotherapies. Addressing these translational barriers may unlock the clinical potential of SOX-targeted approaches to develop more personalized and effective treatment strategies for NSCLC.

## Figures and Tables

**Table I.** SOX factors in the regulation of NSCLC development and progression.

SOX family	SOX member	Role in NSCLC	(Refs.)
B1	SOX1	SOX1 mediates its antitumor effect by directly inhibiting the transcription of HES1.	(77,133)
		SOX1 suppresses tumor cell proliferation and invasion through the downregulation of the Wnt/β-catenin signaling pathway.	
B1	SOX2	SOX2 directly activates the transcription of SLC7A11, thereby promoting cystine uptake and glutathione synthesis, suppressing lipid peroxidation, and enhancing resistance to ferroptosis in tumor cells. Enhances radiation resistance and DNA damage repair ability of cells	(65,86)
C	SOX4	Induces the expression of BMI1, which is involved in NSCLC angiogenesis. Activating CTHRC1 transcriptional activity regulates DNA damage repair and promotes cisplatin resistance in LUAD cells.	(35,134)
D	SOX5	Interaction with YAP1 protein drives the malignant potential of NSCLC cells. Knocking down SOX5 inhibits cell proliferation, migration and EMT progression, and reverses docetaxel resistance in NSCLC. Increases the expression of VEGF and phosphorylation of STAT3, and promotes the tube-forming ability of human umbilical vein endothelial cells.	(49,135)
D	SOX6	miRNA-181b exerts inhibitory effects on tumor cell proliferation and invasion by directly targeting and downregulating SOX6.	(136)
E	SOX9	By upregulating collagen-related genes and promoting extracellular matrix stiffening, SOX9 suppresses dendritic cell infiltration, which indirectly reduces the infiltration and cytotoxic activity of CD8^+^ T and NK cells. Promotes cell proliferation, migration and invasion and regulates β-catenin to promote EMT.	(18,42)
F	SOX7	SOX7 induces mitochondria-dependent apoptosis in cancer cells by activating the P38 signaling pathway and enhancing expression of apoptosis-related genes such as Bcl-2-interacting mediator of cell death	(137)
F	SOX17	miR-200a-3p inhibits the levels of SOX17 and promotes the proliferation and metastasis of NSCLC cells. Upregulation of Wnt signaling by SOX17 methylation promotes NSCLC progression	(15,63)
SOX F	SOX18	SOX18 is upregulated in NSCLC tissues and cell lines, with cytoplasmic expression associated with poor patient outcomes in adenocarcinoma	(54)
H	SOX30	SOX30 suppresses β-catenin expression at the transcriptional level and binds β-catenin to compete with TCF, thereby achieving a dual blockade of the Wnt signaling pathway that effectively inhibits tumor metastasis.. Inhibits the proliferation, migration and invasion of NSCLC cells by activating desmosome gene transcription	(83,138)

NSCLC, non-small cell lung cancer; HES1, hairy and enhancer of split 1; SLC7A11, Solute Carrier Family 7 Member 11; BMI1, B lymphoma Mo-MLV insertion region 1 homolog; CTHRC1, Collagen Triple Helix Repeat Containing 1; LUAD, Lung Adenocarcinoma; EMT, Epithelial-Mesenchymal Transition; NK Cell, Natural Killer Cell; TCF, T Cell Factor.

## Data Availability

Not applicable.
